# Intravitreal Docosahexaenoic Acid in a Rabbit Model: Preclinical Safety Assessment

**DOI:** 10.1371/journal.pone.0096872

**Published:** 2014-05-08

**Authors:** Rosa Dolz-Marco, Roberto Gallego-Pinazo, M. Dolores Pinazo-Duran, Sheila Pons-Vázquez, Joan Carles Domingo-Pedro, Manuel Díaz-Llopis

**Affiliations:** 1 Department of Ophthalmology, University and Polytechnic Hospital La Fe, Valencia, Spain; 2 Ophthalmology Research Unit "Santiago Grisolía", Valencia, Spain; 3 Department of Surgery, Faculty of Medicine, University of Valencia, Valencia, Spain; 4 Biochemistry and Molecular Biology Department, Faculty of Biology, University of Barcelona, Barcelona, Spain; Massachusetts Eye & Ear Infirmary, Harvard Medical School, United States of America

## Abstract

**Purpose:**

The purpose of the present study was to evaluate the retinal toxicity of a single dose of intravitreal docosahexaenoic acid (DHA) in rabbit eyes over a short-term period.

**Methods:**

Sixteen New Zealand albino rabbits were selected for this pre-clinical study. Six concentrations of DHA (Brudy Laboratories, Barcelona, Spain) were prepared: 10 mg/50 µl, 5 mg/50 µl, 2’5 mg/50 µl, 50 µg/50 µl, 25 µg/50 µl, and 5 µg/50 µl. Each concentration was injected intravitreally in the right eye of two rabbits. As a control, the vehicle solution was injected in one eye of four animals. Retinal safety was studied by slit-lamp examination, and electroretinography. All the rabbits were euthanized one week after the intravitreal injection of DHA and the eyeballs were processed to morphologic and morphometric histological examination by light microscopy. At the same time aqueous and vitreous humor samples were taken to quantify the concentration of omega-3 acids by gas chromatography. Statistical analysis was performed by SPSS 21.0.

**Results:**

Slit-lamp examination revealed an important inflammatory reaction on the anterior chamber of the rabbits injected with the higher concentrations of DHA (10 mg/50 µl, 5 mg/50 µl, 2'5 mg/50 µ) Lower concentrations showed no inflammation. Electroretinography and histological studies showed no significant difference between control and DHA-injected groups except for the group injected with 50 µg/50 µl.

**Conclusions:**

Our results indicate that administration of intravitreal DHA is safe in the albino rabbit model up to the maximum tolerated dose of 25 µg/50 µl. Further studies should be performed in order to evaluate the effect of intravitreal injection of DHA as a treatment, alone or in combination, of different retinal diseases.

## Introduction

Polyunsaturated fatty acids (PUFAs) are essential molecules required from de diet, which are classified by the position of the first double bound counting from the metil-terminal in omega-3 -third carbon- (eicosapentaenoic acid, docosahexaenoic acid and α-linolenic acid) and omega-6 -sixth carbon- (linoleic acid and arachidonic acid). The most important omega-3 fatty acid in the nervous system and the retina is docosahexaenoic acid (DHA), particularly highly concentrated in the outer segments of photoreceptors and synaptic membranes. DHA plays a key role in cellular integrity, development and function [1] and it is an essential structural component of cellular membranes. It also shows a functional role, as the proper rhodopsin functionality and visual transduction depend on the adequate proportion of DHA within the retinal cell membranes phospholipids. [2] In addition, DHA displays antiinflammatory properties in contrast to the proinflammatory actions of several members of the omega-6 PUFAs family. [1]

The currently available scientific evidence suggests that DHA may have a protective role against ischemia, oxidative stress and aging in the retina. [3] These effects can result from independent mechanisms such as: membrane functional integrity amelioration, recruitment and up-regulation of anti-apoptotic molecules (as the members of the Bcl-2 gene family) or down-regulation of pro-apoptotic signals, regression or suppression of inflammatory mediators. [4] All this information lead to the design of high-dose DHA oral supplementation trials intending to prevent and to improve the prognosis of retinal degenerative diseases.

The aim of the present study was to evaluate the preclinical safety of intravitreal injection of purified DHA in an albino rabbit model by evaluating functional and anatomical parameters.

## Methods

The study design and experimental protocols were approved by the Ethics and Clinical Research Committee of the Faculty of Medicine of the University of Valencia, (2012) in accordance with the guidelines set forth by the ARVO Statement for the Use of Animals in Ophthalmic and Vision Research and the European Community guidelines regarding animal experimentation.

### Animals

Sixteen adult male New Zealand albino rabbits weighing 2.6 to 3.1 kg were used for the present study. The animals were kept in the University of Valencia in a 12-hour light-dark cycle ambient. Slit-lamp and indirect funduscopic examinations were performed in all animal eyes at baseline. Animals that showed corneal or lens opacity or retinal damage were excluded.

### Intravitreal injection

The drug used for the assays was DHA (purity of 70% as triglycerides as measured by high-performance liquid chromatography and thin-layer chromatography) obtained from Brudy Laboratories (Barcelona, Spain). The fatty acid profiles of nanoemulsions used in the present study are expressed in Table 1. DHA was administered as nanoemulsions diluted with balanced saline solution to obtain six different concentrations: 10 mg/50 µl, 5 mg/50 µl, 2.5 mg/50 µl, 50 µg/50 µl, 25 µg/50 µl, 5 µg/5 µl. Each concentration was injected intravitreally in the right eye of two animals.

**Table 1:** Fatty acid profiles of nanoemulsions used.

Fatty acids	Mol%
Total SFA	2,3±0,4
Total MUFA	3,2±0,3
Total PUFA	94,4±2,0
Total Omega-6	4,9±1,8
Total Omega-3	89,6±1,5
C22∶6n-3 (DHA)	88,3±1,8
n-6/n-3 Ratio	0,05±0,1

**Abbreviations:** Total SFA, Total saturated fatty acids; Total MUFA, Total monunsaturated fatty acids; Total PUFA, Total polyunsaturated fatty acids; DHA, docosahexaenoic acid. Data are given as mean ± SD of mol% of fatty acid.

We performed the study in two periods. Firstly we used high doses of intravitreal DHA in order to define the maximal tolerated dose. Thereafter we used corrected DHA concentrations in order to establish the range of optimal doses administered as a single dose injection.

During the first period of the study, 8 rabbits were used. A control group of 2 rabbits (Group 1) was injected intravitreally with 0.05 ml of the vehicle solution (saline solution); other 3 groups of 2 animals each were injected intravitreally with different concentrations of intravitreal DHA (group 2 received 0.05 ml containing 2.5 mg of DHA; group 3 received 0.05 ml containing 5 mg of DHA; and group 4 received 0.05 ml containing 10 mg of DHA).

During the second period of the study, we used 8 rabbits. A control group of 2 rabbits (Group 5) was injected intravitreally with 0.05 ml of the vehicle solution (saline solution); other 3 groups of 2 animals each, were injected intravitreally with different concentration of intravitreal DHA (group 6 received 0.05 ml containing 5 µg of DHA; group 7 received 0.05 ml containing 25 µg of DHA; and group 8 received 0.05 ml containing 50 µg mg of DHA).

The rabbbits were anesthetized with a mixture of ketamine hydrochloride (50 mg/ml) and xylazine hydrochloride (5 mg/ml). Topical anaesthesia was applied using oxibuprocaine hydrochloride 1 mg/ml and tetracaine hydrochloride 4 mg/ml (Colircusi Anestesico Doble, Alcon laboratories, Barcelona, Spain). Diluted povidone-iodine solution was applied before and after injection. Intravitreal injections were performed using a 32-gauge needle attached to a syringe inserted 2 mm posterior to the limbus in the inferior temporal quadrant. All rabbits were treated with daily application of fluoroquinolone ointment (ciprofloxacine) after the injection as antibiotic prophylaxis.

Slit lamp and indirect funduscopic examinations were performed in all eyes immediately after the injections and after 1 week. We evaluated changes in the cornea, lens, vitreous, retina and optic nerve.

### Electroretinogram

The right eyes of all animals were evaluated by electroretinography (ERG) at baseline and 1 week after the injection. After inducing mydriasis with 1 drop of tropicamide (1%), the animals were dark-adapted for at least 1 hour, and then standard full-field ERGs were recorded using a corneal contact lens ERG-jet electrode (MicroComponents, UniversoPastique, Switzerland). The reference and ground electrodes were stainless surgical needles (NeuroLine, Ambu Lab, Ballarup, Denmark), that were placed subcutaneously at the forehead (ground) and ear (reference). The ERG signals were recorded using the hand-held portable ERG system EPH-01 (Ephiós ab, Rejmyre, Sweden). The dark-adapted scotopic response (rod response) and the scotopic response (maximal response, cone and rod) were recorded. Flash intensity was 1.7 cd-s/m-2 and acquisition time was 200 msec for each registration. The amplitudes of A- and B-waves were calculated at baseline and 1 week after the injection. ERG changes were considered significant if the differences in amplitude (A- and B-waves) from baseline to the final values were higher than 30%. [5]. ERG data were interpreted and interferences were automatically removed with the software of EPH-01 1.2 version (Ephiós ab, Rejmyre, Sweden).

### Clinical Examination

Eyes were clinically examined at baseline, immediately before the injection and one week after the injection. Pupils were dilated with tropicamide eye drops. The following parameters were recorded through the slit lamp examination: corneal transparency, conjunctival appearance, anterior chamber, iris and lens status, and ocular fundus characteristics.

All eyes were examined by indirect ophthalmoscopy with a 20 D lens for imaging of the retina in order to exclude possible diseases affecting the vitreous, retina and choroid.

### Histological Examination

One week after the intravitreal injection the animals were euthanized with a lethal overdose of endovenous pentobarbital (100 mg/kg) into the ear vein. Drops of physiological saline were applied to the eyes to prevent drying. Aqueous humour and vitreous samples were obtained, deposited in labelled Eppendorf tubes (500 µl), frozen at −80°C and stored until processing. Immediately, the eyes were operated under a dissection binocular microscope. Each eye was fixed with angled forceps to cut away the conjunctiva by Wescott scissors. The extraocular muscles and the retrobulbar optic nerve attached to the globe were cut and the eyeball was lifted from the orbit. The eyeball was rinsed in PBS. Then the globe was inmovilized and the sclera was incised 2 mm posterior to the limbus to facilitate fixation with 10% buffered formalin. After 72 hours, the eyes were sectioned at the retro-equatorial level to obtain small pieces of the retina that were oriented and separately dehydrated in a series of 95% ethanol, and embedded in paraffin. Retinal trans-sections of 5-μm thickness were collected on poly-L-lysine–coated microscopic slides for H&E staining and Masson trichrome, and the slides were examined by light microscope (BX-50; Olympus, Tokyo, Japan) and photographed with a digital video camera (DP-71 CCD; Olympus). [6]

A morphologic examination of the retina was performed evaluating the loss of cellular elements, cell disorganization, apoptotic cells or necrotic degeneration. Morphometric analyses were performed on the retinal trans-sections to evaluate the total retinal thickness and layering (ganglionar cell layer, inner plexiform layer, inner nuclear layer, outer plexiform layer and outer nuclear layer/photoreceptor layer) and the density of stained nuclei within the ganglion cell layer, which was determined in the vicinity of the optic disc, where the density of these cells is more stable and changes little with direction or eccentricity. [7]

### Aqueous and vitreous humor samples analysis

Concentrations of the principal fatty acids in the aqueous and vitreous humor samples were measured by gas chromatography in the laboratory of chemistry of the University of Barcelona (Barcelona, Spain).

### Statistical analysis

We analyzed the results of the different studied parameters by the SPSS 21.0 (IBM Corp). Non-parametric tests (Kendall’s Tau and Spearman’s Rho) were used to perform the correlation of the different DHA doses and the electroretinography and histological parameters. In the comparison of independent variables, we used the Mann Whitney’s U test and for the dependent variables we analyzed the data with Wilcoxon test.

## Results

### Clinical Examination

**Figure 1:**
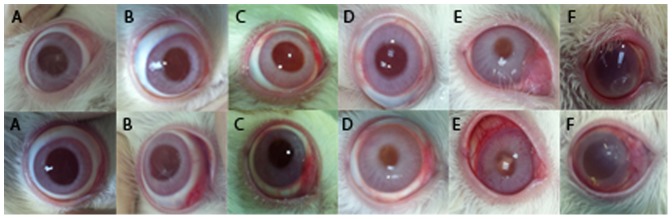
Color photographs of the eyes one week after intravitreal injection of 0.05 ml of different concentrations of DHA. Eyes injected with 5 µg (A), 25 µg (B), and 50 µg (C) of DHA showed no inflammatory signs in the anterior segment. Eyes injected with the higher concentrations of DHA showed ocular inflammatory signs directly proportional to the injected concentration. The eyes injected with 2.5 mg (D) and 5 mg (E) of DHA showed mild to moderate inflammation in the anterior chamber, while the eyes injected with 10 mg (F) of DHA demonstrated a severe ocular inflammation. In one case (F, top row) a corneal ectasia appeared associated with surrounding corneal edema.

No complications directly related to the intravitreal injection were evidenced (cataract, vitreous haemorrhage or retinal detachment). Color photographs of the eyes are shown in Figure 1.

In the first period of the study, slit lamp examination revealed a significant ocular inflammation in the rabbits included in group 4 (10 mg/50 µl), with one case of acute corneal melting with ectasia and mild surrounding edema. This was managed with ciprofloxacine ointment in order to avoid any misinterpretation of the inflammation. Through the follow-up the inflammation decreased and the corneal melting resolved with a final corneal opacity. A mild-moderate perikeratic conjunctival hyperaemia was also remarkable in groups 2 and 3 directly proportional to the injected concentration. Thus, concentrations of DHA used in groups 2, 3 and 4 (2’5 mg/50 µl, 5 mg/50 µl and 10 mg/50 µl respectively) were considered clinically unsafe.

Slit lamp examination in the second period of the study in groups 6, 7, 8 was unremarkable, with no signs of inflammation and no corneal, lens or vitreous opacity. In addition, no clinical manifestations were observed in control groups (groups 1 and 5). No signs of retinal necrosis or cystic degeneration were observed in any of the groups.

### Electroretinogram

No statistically significant changes in A- and B- waves amplitudes measured in scotopic and photopic conditions were observed comparing ERG data at baseline (before injection) and 1 week after intravitreal injection of the different concentrations of DHA (Data are summarized in Table 2). In addition, post-injection ERG study did not show significant differences between control eyes and DHA-injected eyes (Table 3). There was no statistically significant correlation between the DHA injected dose and the post-injection ERG data according the Kendall’s Tau tests (Scotopic A-wave p = 0,906; Scotopic B-wave p = 0,887; Photopic A-wave p = 0,256 and Photopic B-wave p = 0,152).

**Table 2:** Comparison of baseline and 1 week post-injection ERG parameters (mV).

	2		3		4	
	mean ± SD	P value	mean ± SD	P value	mean ± SD	P value
PRE-POST Scotopic A-wave	−189,60±6,56/−193,46±5,07	0,416	−185,42±4,96/−185,22±5,51	0,686	−182,44±4,85/−184,58±4,71	0,686
PRE-POST Scotopic B-wave	333,80±6,45/331,52±4,04	0,345	332,02±3,52/333,42±6,59	0,892	327,18±6,16/323,56±8,55	0,225
PRE-POST Photopic A-wave	−146,32±1,71/−142,08±3,82	0,043	−143,86±0,98/−139,50±2,27	0,043	−137,10±6,19/−136,92±2,14	0,5
PRE-POST Photopic B-wave	102,30±1,12/101,22±1,40	0,043	100,66±1,53/100,18±1,02	0,5	99,08±2,34/97,94±2,27	0,465

	6		7		8	
	mean ± SD	P value	mean ± SD	P value	mean ± SD	P value
PRE-POST Scotopic A-wave	−192,62±4,35/−185,22±5,08	0,08	−188,98±5,06/−188,54±7,02	0,893	−188,60±8,49/−189,30±5,08	0,893
PRE-POST Scotopic B-wave	334,06±3,27/321,12±8,26	0,08	323,30±4,94/326,72±10,22	0,686	324,92±10,65/327,46±7,54	0,893
PRE-POST Photopic A-wave	−143,52±4,40/−136,80±5,09	0,08	138,88±4,92/−140,36±6,06	0,786	−138,00±7,33/−140,52±4,99	0,5
PRE-POST Photopic B-wave	101,64±1,99/98,60±2,65	0,08	99,32±2,32/99,48±3,68	0,684	98,22±3,36/100,52±2,42	0,225

**Table 3:** Comparison of ERG parameters (mV) between control groups and DHA injected groups.

	1	5	2		3		4	
	mean ± SD	mean ± SD	mean ± SD	P value	mean ± SD	P value	mean ± SD	P value
Scotopic A-wave	−188,02±6,69	−186,06±7,99	−193,46±5,07	0,075	−185,2±5,51	0,713	−184,58±4,71	0,668
Scotopic B-wave	331,30±9,36	331,24±8,96	331,52±4,04	0,854	333,42±6,59	0,624	323,56±8,55	0,27
Photopic A-wave	−144,30±5,84	−142,24±7,00	−142,08±3,82	0,462	−139,5±2,27	0,198	−136,92±2,14	0,111
Photopic B-wave	101,22±2,46	100,84±3,32	101,22±1,40	0,624	100,18±1,02	0,111	97,94±2,27	0,066

	1	5	6		7		8	
	mean ± SD	mean ± SD	mean ± SD	P value	mean ± SD	P value	mean ± SD	P value
Scotopic A-wave	−188,02±6,69	−186,06±7,99	−185,22±5,08	0,357	−188,54±7,02	0,806	−189,30±5,08	0,22
Scotopic B-wave	331,30±9,36	331,24±8,96	321,12±8,26	0,055	326,72±10,22	0,391	327,46±7,54	0,391
Photopic A-wave	−144,30±5,84	−142,24±7,00	−136,80±5,09	0,086	−140,36±6,06	0,391	−140,52±4,99	0,5
Photopic B-wave	101,22±2,46	100,84±3,32	98,60±2,65	0,111	99,48±3,68	0,391	100,52±2,42	0,713

### Histological Examination

**Figure 2:**
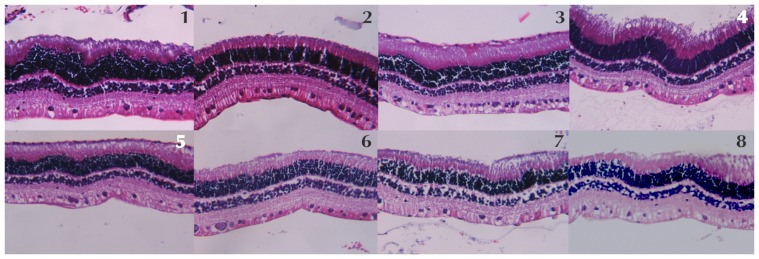
The light microscopy histological examination of each group, labeled on top of each image, did not show any sign of retinal disarrangement, cellular apoptosis or ischemia.

The retinal morphologic examination with light microscopy did not evidence any retinal pigment epithelium or neurosensory retina abnormality in the eyes of rabbits injected with the different concentrations of DHA compared with control rabbits (injected with saline solution). No disarrangement of photoreceptors was evidenced. There were no signs of cellular apoptosis or ischemia (Figure 2).

The morphometric parameters analyzed and the statistical analysis are summarized in the Table 4. The morphometric analysis did not reveal any statistically significant correlation between the DHA injected dose and the different retinal thickness measures according with Kendall’s Tau tests (total retinal thickness, p = 0.892; ganglionar cell layer, p = 0.136; inner plexiform layer, p = 0.828; inner nuclear layer p = 0.836; outer plexiform layer p = 0.942; outer nuclear layer-photoreceptors p = 0.705). However the Mann-Whitney’s U test evidenced statistically significant differences between the control groups (1 and 5) and group 8 in the total retinal thickness (p>0.001), the outer plexiform layer thickness (p = 0.024), and the outer nuclear layer/photoreceptor thickness (p = 0.018). Further comparisons between the different retinal measures of DHA injected eyes and control eyes did not evidenced any other significant differences for the groups 2, 3, 4, 6 and 7.

**Table 4:** Comparison of mean retinal thickness measures (microns) between control group and DHA injected groups.

	2		3		4		6		7		8	
Retinal thicknes measures (microns)	mean ± SD	P value	mean ± SD	P value	mean ± SD	P value	mean ± SD	P value	mean ± SD	P value	mean ± SD	P value
Total retinal thickness	168,7 ± 8,42	0,092	177,76±11,51	0,673	187,4±7,61	0,303	192,72±34,26	0,851	170,82±13,50	0,261	147,48±6,23	>0,001
Ganglionar cell layer	29,12±7,29	0,512	25,42±6,62	0,111	29,57±3,84	0,512	28,27±6,22	0,349	31,39±1,74	0,708	26,38±2,05	0,223
Inner plexiform layer	29,06±3,85	0,708	31,00±4,35	0,261	31,51±8,53	0,779	29,05±4,31	0,574	25,78±5,15	0,64	25,78±5,15	0,092
Inner nuclear layer	22,83±2,75	0,888	19,54±3,13	0,061	26,61±2,74	0,068	24,66±1,48	0,303	22,22±3,68	0,542	20,61±4,84	0,068
Outer plexiform layer	7,36±1,00	0,779	8,08±1,70	0,426	7,68±1,00	0,673	7,81±1,60	0,925	8,79±2,60	0,399	6,14±0,88	0,024
Outer nuclear layer-photoreceptors	75,48±13,15	0,111	78,53±6,18	0,134	91,91±9,79	0,512	80,89±16,48	0,454	76,52±4,14	0,075	67,05±11,7	0,019

The mean density of stained ganglionar cell nuclei did not showed any statistically significant correlation between the DHA injected dose (p = 0,784). No statistically significant differences in the number of ganglionar cell nuclei between control eyes and DHA-injected eyes were evidenced (Table 5).

**Table 5:** Comparison of mean number of stained ganglionar cell nuclei between control group and DHA injected groups.

Group	1	5	2	3	4	6	7	8
Mean number of stained ganglionar cell nuclei.	32	39	37,5	29	35	38	20,5	19,5
p value	NA	NA	0,44	0,99	0,99	0,99	0,12	0,12

### Aqueous and vitreous humor samples analysis

Concentrations of the principal fatty acids in the aqueous and vitreous humor samples of eyes treated with DHA compared with control eyes are summarized in Table 6. We found statistically significant differences in the concentration of DHA in vitreous samples between the control group and all the DHA-injected groups. In the aqueous humor samples we only found statistically significant differences between the control group and group 4 (which was injected with the higher dose of DHA: 10 mg/50 µl).

**Table 6:** Fatty acid profiles in total lipid of vitreous and aqueous humor samples.

Fatty acids in vitreous humor samples	Control	Intravitreal DHA Supplemented					
		5 µg	25 µg	50 µg	2,5 mg	5 mg	10 mg
Total SFA	39,5±1,4	39,7±0,4	40,6±2,0	36,4±1,5	25,1±3,1†	11,8±2,4†	8,4±0,3†
Total MUFA	44,9±2,7	45,3±0,2	43,4±1,2	39,8±1,9	27,1±1,5†	14,4±2,5†	12,3±0,6†
Total PUFA	14,2±1,0	17,1±1,8	17,8±1,6	20,2±3,3	44,1±6,3†	67,9±5,3†	77,4±2,7†
Total Omega-6	9,3±1,0	10,4±2,2	9,5±1,2	8,7±2,5	7,0±1,2	5,6±1,2	7,1±0,6
C18∶2n-6 (LA)	4,8±0,6	4,9±1,9	4,2±1,3	3,4±0,4†	2,0±0,4†	1,5±0,2†	1,9±0,3†
C20∶4n-6 (ARA)	2,2±0,3	2,9±0,5	3,7±1,1	4,0±1,1	2,5±0,5	2,0±0,6	1,9±0,7
Total Omega-3	4,9±0,6	6,7±0,4†	8,3±0,4†	15,1±0,8†	37,1±4,4†	62,4±7,8†	70,3±6,1†
C18∶3n-3 (ALA)	0,8±0,6	0,8±0,4	0,7±0,1	1,0±0,4	0,3±0,4	0,2±0,1	0,2±0,1
C20∶5n-3 (EPA)	0,5±0,1	0,6±0,1†	0,8±0,1†	1,0±0,1†	3,0±0,8†	5,4±0,8†	6,5±0,9†
C22∶5n-3 (DPA)	0,8±0,1	1,5±0,6	2,5±0,6†	4,4±1,1†	7,7±2,1†	9,8±0,2†	10,9±2,3†
C22∶6n-3 (DHA)	2,6±0,1	3,8±0,3†	4,2±0,2†	8,5±0,8†	26,0±3,4†	46,8±7,0†	55,1±7,1†
n-6/n-3 Ratio	1,9±0,3	1,5±0,4	1,2±0,1†	0,8±0,2†	0,2±0,1†	0,1±0,1†	0,1±0,1†

Fatty acids in aqueous humor samples	Control	Intravitreal DHA Supplemented					
		5 µg	25 µg	50 µg	2,5 mg	5 mg	10 mg
Total SFA	34,7±0,4	35,2±1,3	34,5±0,5	31,2±0,9†	32,0±1,1†	32,2±0,6†	32,1±1,5†
Total MUFA	27,6±0,8	28,7±0,9	28,6±0,4	28,8±0,7	29,5±1,6	30,2±1,0†	30,3±0,4†
Total PUFA	37,6±2,0	37,2±2,1	39,7±1,1	38,0±1,6	36,7±2,5	39,9±1,2†	40,7±2,7†
Total Omega-6	18,9±1,8	18,2±0,1	20,6±0,7	18,5±0,4	18,7±0,7	20,2±2,6	18,6±1,7
C18∶2n-6 (LA)	11,3±0,5	11,6±2,0	10,9±1,8	11,6±1,9	10,8±1,8	12,4±1,6	11,5±0,3
C20∶4n-6 (ARA)	7,4±0,1	6,6±0,1	9,1±1,1	6,9±0,1	7,9±0,1	7,8±0,4	7,1±0,3
Total Omega-3	18,7±0,5	19,1±2,0	19,1±1,8	19,5±1,8	18,0±1,8	19,7±1,3	22,1±1,1†
C18∶3n-3 (ALA)	2,5±0,4	2,6±0,1	2,4±0,6	2,7±0,4	2,2±0,2	2,7±0,1	2,2±0,3
C20∶5n-3 (EPA)	0,2±0,2	0,2±0,3	0,2±0,3	0,6±0,3	0,2±0,3	0,4±0,3	0,7±0,4
C22∶5n-3 (DPA)	12,4±1,0	12,8±0,5	12,5±0,7	12,2±0,6	11,5±0,9	12,0±0,9	12,7±0,7
C22∶6n-3 (DHA)	3,7±0,8	3,5±1,1	4,0±0,8	4,0±1,3	4,0±0,9	4,6±0,3	6,5±0,3†
n-6/n-3 Ratio	1,0±0,1	1,0±0,1	1,1±0,1	0,9±0,1	1,0±0,1	1,0±0,1	0,8±0,1

Data are given as mean ± SD of mol% of fatty acid. For each fatty acid, values with symbol (†) are significantly different (p<0,05). **Abbreviations:** Total SFA (saturated fatty acids); Total MUFA, (monunsaturated fatty acids); Total PUFA (polyunsaturated fatty acids); ARA, (arachidonic acid); DPA (docosapentaenoic acid); EPA (eicosapentanoic acid); LA (linoleic acid); ALA (alpha linolenic acid); DHA (docosahexaenoic acid).

## Discussion

The retina is extremely susceptible to oxidative damage. In contrast, it is well known the essential role of omega-3 PUFAs in maintaining the retinal function and integrity and there are several reports of the protective role against oxidative stress. [1]–[4], [8] Furthermore the loss of DHA-rich photoreceptors cells in the retina constitutes a hallmark of different retinal degenerations such as age-related macular degeneration (AMD), retinitis pigmentosa, Stargardt disease or glaucoma. [9] A number of epidemiological studies have suggested that lower dietary intakes and lower circulating concentrations of DHA are associated with higher risk of AMD, [10] whereas other reports have shown lower levels of DHA in the plasma and the erythrocytes of patients with retinitis pigmentosa [11] or primary open angle glaucoma. [12]–[13] These data led to the design of several dietary supplementation trials including DHA for AMD, [14]–[15] Stargardt disease, [16] Best’s maculopathy [17] and retinitis pigmentosa [18]–[19] and to the commercialization of DHA-rich dietary supplements with the aim of maintaining or increasing retinal DHA concentrations in the retina. These studies reported the efficacy of supplementation in AMD [13]–[14] and retinitis pigmentosa, [18] and showed a remarkable influence in some functional parameters, albeit no benefit was achieved with the short-term treatment for Stargardt’s disease [16] or Best disease. [17]

Beneficial effects of DHA supplementation are significant in patients with AMD. Even when AMD does not lead to blindness, there is a strong negative impact on the independence and the quality of life. [20] Despite the magnitude of this problem, very few factors have been identified as capable to modify the natural course of AMD once signs of early -small-intermediate hard drusen (≤ 124 µm) and/or retinal pigment epithelium (RPE) changes- or intermediate –some large soft drusen (>124 µm) and/or geographic atrophic patches without central macular involvement- AMD appear.

To the best of the authors’ knowledge, the safety of intravitreal injection of purified DHA has not previously been reported. We have used six different concentrations of purified DHA. Due to the mild-moderate inflammatory reaction induced directly proportional to the concentration of DHA we considered unsafe the concentrations of 10 mg/50 µl, 5 mg/50 µl, 2.5 mg/50 µl. Thus we corrected the doses of purified DHA in a second phase of the study and the results of our study suggest that DHA may be a safe intravitreal drug in the rabbit model up to 25 µg/50 µl, with no evidence of toxicity in the slit-lamp examination, ERG data and light microscopy histologic analysis. The maximal tolerated dose was 0.05 ml of 25 µg/50 µl DHA. The safe dose range of DHA in the present study was 5 µg/50 µl to 25 µg/50 µl.

We hypothesize that the intravitreal administration of DHA into the vitreous may be a potential therapy to prevent the progression of degenerative retinal diseases by restoring the high concentration of DHA in the retina and modulating different homeostatic mechanisms. The limitations of our study to draw final conclusions about the safety of intravitreal DHA include: the short number of rabbits used, the differences between the rabbit and the human retinas and the restrictions in the functional tests subsequent of working with an animal model.

In conclusion, this preliminary study suggests that intravitreal DHA is safe up to 25 µg/50 µL in the rabbit eye. Further studies are warranted to evaluate the safety and efficacy of intravitreal DHA in human eyes.
